# Embedding digital sleep health into primary care practice: A triangulation of perspectives from general practitioners, nurses, and pharmacists

**DOI:** 10.1177/20552076231180970

**Published:** 2023-06-23

**Authors:** Janet MY Cheung, Zoe Menczel Schrire, Melissa Aji, Matthew Rahimi, Helena Salomon, Iliana Doggett, Nicholas Glozier, Delwyn J. Bartlett, Keith Wong, Ronald R. Grunstein, Christopher J. Gordon

**Affiliations:** 1 Faculty of Medicine and Health, School of Pharmacy, 4334 The University of Sydney, Sydney, Australia; 2 Healthy Brain Ageing Program, The University of Sydney School of Psychology, Sydney, Australia; 3 Black Dog Institute, University of New South Wales, Sydney, Australia; 4 CIRUS, Centre for Sleep and Chronobiology, 104349 The Woolcock Institute of Medical Research, Syndey, Australia; 5 Faculty of Medicine and Health, Central Clinical School, 4334 The University of Sydney, Sydney, Australia; 6 Faculty of Medicine and Health, Sydney Nursing School, 4334 The University of Sydney, Sydney, Australia

**Keywords:** Digital health, sleep health, insomnia, mixed methods, primary care, general practitioner, pharmacist, community practice nurse, Australia

## Abstract

**Introduction:**

While digital health interventions (DHIs) can potentially address the unmet needs for sleep health services, little is known about their implementation in practice. The current study aimed to explore primary care health providers’ attitudes and beliefs towards DHIs for sleep and implementation into practice.

**Methods:**

A cross-sectional online survey was administered to Australian primary care health professionals: general practitioners (GPs), community nurses, and community pharmacists. Semi-structured interviews were conducted within a sub-sample of participants exploring their experiences with DHIs and perceived barriers/facilitators for embedding DHIs into primary care. Semi-structured interviews were thematically analysed using the framework approach to contextualise survey findings.

**Results:**

Ninety-six surveys were returned (GPs  =  36, nurses = 30, and pharmacists = 30) and 45 interviews conducted (GPs  =  17, nurses = 14, and pharmacists  =  14). From the survey, GPs were more likely to endorse familiarity (*p*  =  0.009) and use (*p* < 0.001) of sleep DHIs in clinical practice than pharmacists and nurses. GPs were more interested in utilising the diagnostic features within a sleep DHI (*p*  =  0.009) compared to other professionals. Thematic analysis of the interviews revealed three major themes, contextualised by profession: (1) *Scope for DHIs in Current Practice*, (2) *Practice Gaps and Training Needs,* and (3) *Envisioning a Model of Care Using Sleep DHIs*. While DHIs can potentially improve care, greater clarity of care pathways and reimbursement structures are needed for integration into practice.

**Conclusion:**

Primary care health professionals highlighted the training, care pathway and financial models required to realise the potential for translating findings from efficacy studies for DHIs into primary care to optimise sleep health.

## Introduction

Sleep disturbances are highly prevalent in primary care but are sub-optimally addressed.^1,2^ Among factors such as lack of time, workload, and reimbursement, is the shortage of sleep physicians and psychologists to meet the demand for specialist sleep health services.^2,3^ Given sleep medicine is not a procedure-based speciality, it is amenable to digitalisation. As such, a multitude of digital health interventions (DHIs) have been developed to support core areas of sleep medicine practice over the last decade. Technological advances have further created new platforms for patient care access, ranging from telehealth video-conference calls that replace in-person clinic visits^4^ to standalone web-based programs that offer patients on-demand therapeutic interventions. Specifically, sleep-related DHIs have targeted the treatment of sleep disorders such as obstructive sleep apnea using automated feedback mechanisms to improve adherence to continuous positive airway pressure (CPAP) therapy,^5^ and delivering cognitive behavioural therapy for insomnia (CBT-I) digitally.^6^ The latter has gained significant traction where several landmark studies and systematic reviews have demonstrated the feasibility and efficacy for digitalised CBT-I albeit smaller effect sizes when compared to face-to-face therapy (Cohen’s d  =  1.2 vs. Cohen's d  =  2.3),^7^ while potentially overcoming geographical,^8^ resource and scheduling constraints of traditional care models.^6,9,10^ A recent meta-analysis has further demonstrated comparable effects between face-to-face CBT-I and digital CBT-I involving a virtual or real therapist,^11^ highlighting the potential for delivering digitalised CBT-I with guidance from a primary care health professional.^6,8–10^ More broadly, sleep telehealth services have also been evaluated in a number of settings in the United States, showing great promise for remote care^12^ and are increasingly included as part of formalised training.^13^

While the empirical evidence is promising, a considerable gap exists in understanding the real-world effectiveness of DHIs for sleep, or how to implement and embed them into routine primary care practice where their scalability is most useful. Effectively implementing sleep DHIs in primary care and the broader community is a timely and pertinent issue for public health policy, given the socio-economic impacts attributable to poor sleep,^14^ which are further worsened by the effects of COVID-19 on sleep.^15,16^ More immediately, primary care providers may have renewed interest in deploying DHIs in their practice as many are likely to be already operating in an increasingly digitalised workspace.^17^ Longer term, there is a need to strategically plan and build capacity for the anticipated increase in patient demand for specialist sleep services given the bi-directional relationship between sleep and mental health,^18^ that is exacerbated post-pandemic.^19^ To date, much of the literature on the implementation of digital health technologies in primary care has focused on the perspective of general practitioners (GPs) despite the diversity of professional stakeholders in primary care including nurses and community pharmacists.^20,21^ From a translational perspective, this represents a major gap in sleep medicine, considering the range of primary care providers that patients initially seek help from.^22^ Further, the digitalisation process in the Australian practice context has largely focused on embedding systems into practice such as My Health Record and electronic prescribing.^23,24^ In contrast, little is known about the factors/constraints influencing the uptake, scalability, and dissemination of digital therapeutics for managing disease, despite their role in the next key phase for digitalisation in the healthcare landscape.

As such, the present study aims to capture the perspectives of GPs, community nurses, and community pharmacists with respect to their experiences and attitudes towards implementing digital health technologies for improving sleep health. More specifically, this study sought to gain deeper insight into the barriers and enablers of implementation for the respective professional disciplines.

## Methods

For the purpose of this study, DHIs were operationalised as ‘the intersection of health care with the internet in which wearable devices, information technology (IT) and electronic communication tools converge to support the practice of medicine’.^25^ This definition was also used to guide the framing of our research questions, study design, and analysis.

A mixed methods study was conducted using an online survey instrument and a series of semi-structured interviews conducted with GPs, community nurses, and community pharmacists practising in Australian primary care settings. The research protocol and study materials were approved by the Sydney Local Health District Human Research Ethics Committee (Approval No. 2019/ETH 1330).

### Participants and recruitment

To capture the perspectives of key professional stakeholders in primary care, we recruited a convenience sample of individuals who were practising GPs, community nurses, and community pharmacists. Appreciating the heterogeneity of primary care practice, we sought to capture a representative sample of participants across the three professions, with a broad range of practice experience, that is, duration of practice and location of practice (i.e., rural vs. metropolitan).

Participants were recruited between August 2020 and December 2021 through active snowballing, reaching out to Australian public health networks, and drawing on the professional networks of the research team. Social media platforms (i.e., Twitter and Facebook) of the respective professions were also used to reach out to prospective participants. Participants responded to a targeted advertisement flyer on DHIs for sleep which contained an embedded study survey link and a quick response (QR) code. We note that the recruitment period of this study also coincided with the height of the COVID-19 pandemic in Australia. During this period, various public health measures and restrictions took place in local government areas, necessitating rapid digitalisation in healthcare delivery.

Prospective participants who were interested in participating clicked on a link/QR code and were taken to a landing page of the study website which contained a participant information sheet. Consenting participants completed the online survey instrument and on completion were asked about their interest in being interviewed. Those interested were requested to leave their contact details, and an interview on Zoom (audio only) or telephone was organised by a member of the research team. Informed consent was verbally verified prior to commencing the interview. Upon completion of the interview, participants received a $50 gift card as compensation for their time. Those who only completed the survey went in a draw to win one of three $200 gift cards. As this was an exploratory study, with the goal of measuring group differences we adopted a rough estimate of 30 participants per cell corresponding to each of the professions which equates to an approximate total sample size of 90.^26^ Recruitment for the qualitative interviews continued until thematic saturation was reached where additional interviews do not yield any new thematic categories.^27^ We estimated that approximately 12 participants from each professional group would yield thematic saturation and adopted a stop criterion of two consecutive interviews not yielding new thematic categories.^27,28^

## Research instruments

### Online survey

An online survey was developed to capture participant demographics, practice context, and current experience with using DHIs. A 10-item digital health implementation instrument was adapted from the items originally developed by Leigh and Ashall-Payne^29^ to capture the importance of core attributes for participants to consider when implementing DHIs in practice (e.g. data security, privacy, and legal compliance). Each item was scored on a 7-point Likert scale (0 = not at all important, 6 = extremely important), but the instrument does not have a cut-off score for participants’ global ratings of the items (Supplemental Appendix A). Based on the modified Monash model,^30^ participants’ geographical location of practice was recoded into three categories for ease of interpretation: metropolitan (MM1), regional (MM2), and rural/remote (MM3 to MM7). Participants also indicated their interest to be interviewed.

### Semi-structured interviews

The interviews sought to capture participants’ practice environment, experiences with DHIs, and attitudes towards embedding DHIs targeting sleep health at their respective practice. As part of the interview, participants also described the features and functions of what they considered an ideal sleep DHI. Enablers and barriers to DHI implementation were also explored. The interview guide was developed by the first author (pharmacist academic) in collaboration with a nurse academic (CG) and clinical psychologist and DHI expert (MA). The included questions (Supplemental Appendix B) were largely informed by the literature on the management of sleep health^31^ and the implementation/uptake of digital health in primary care.^32,33^ The interview guide was first piloted with a pharmacist, GP, and nurse known to the researchers and feedback was used to further refine the documents, but these interviews were not included in the final analysis. Subsequent interviews were conducted with participants via Zoom or telephone by two researchers: ZMS and JC. All interviews were digitally recorded and transcribed verbatim by an independent transcriber and checked for fidelity with the recorded audio by ZMS. Field notes were taken to facilitate analysis.

### Statistical analysis

Descriptive statistics were computed for demographic and practice variables. Mean score ratings of the relative importance for each of the 10 attributes (e.g., financial incentives, data security, and cost to patients) for implementing DHIs were also computed for the total sample and by the health profession. To explore differences between the three professions in implementing DHIs, each was treated as an independent variable. Associations between categorical variables and profession were first explored using the chi-square test (e.g. use of DHIs in current practice; yes/no). The Benjamini–Hochberg procedure was used to adjust *p*-values to control for false discovery rates in multiple comparisons with the critical value set at 0.25. A one-way analysis of variance was used to analyse continuous variables (e.g. perceived importance of data security). Post-hoc pairwise comparisons were conducted using the Tukey test, which computes the minimum difference between mean values to identify significant differences. Column proportions were compared to identify differences between groups. For missing data (rate: 2%), a pairwise deletion was used to maximise the data available.

### Qualitative data analysis

The framework approach (FA) was adopted to guide the study design and analysis, facilitated by QSR NVivo 12 software.^34^ FA evolved out of an applied social policy research tradition with the goal of meeting specific information needs and developing potential actionable outcomes in set timeframes.^35,36^ Additionally, FA merges inductive and deductive qualitative research traditions, addressing a priori questions set at the beginning of the study while identifying emergent ideas raised by the participants.

FA can be broadly divided into five key steps: **Familiarisation** of data through reading interview transcripts and field notes iteratively to understand the concepts and issues raised by the participant. This is followed by **Identifying a thematic framework** by integrating a priori issues set out by the schedule of questions with key ideas raised by individual respondents during the familiarisation stage. **Indexing** involves the application of the thematic framework to sift through individual transcripts systematically to identify relevant sections of text corresponding to the relevant thematic sections of the framework (i.e. assigning a code). **Charting**, which involves extracting and summarising the indexed portions of text to reorganise the material into thematic matrices with headings and subheadings containing related thematic categories and results in multiple charts. Each case is also clearly labelled for identification purposes during analysis. **Mapping and interpretation** are the final stages of analysis where the phenomenon under study is described and explained by exploring within-case and cross-case associations that lead to the identification of emergent themes.

Participant quotes were selected to illustrate the identified themes. To maintain confidentiality, each participant was assigned a code, with the letter P followed by a number to indicate interview order, their professional discipline (i.e. GP, nurse, and pharmacist), gender (M: male; F: female), age, and years of practice.

## Results

### Participant characteristics

In total, 96 participants completed the survey comprising 36 GPs, 30 nurse practitioners, and 30 pharmacists. The mean age of all participants was 41.91 years (SD  =  13.34). Our participant sample comprised approximately 70% female (*n*  =  67) and 30% male (*n*  =  29). Of the total sample, 46.9% (*n*  =  45) were interviewed. Table 1 summarises participant characteristics as a whole and by profession.

**Table 1.** Summary of participant characteristics by profession.

	Total sample (*n* = 96)	General practitioner (*n* = 36) mean ± SD	Community nurse (*n* = 30) mean ± SD	Community pharmacist (*n* = 30) mean ± SD	*p*-value
Age	41.9 ± 13.3	48.6 ± 12.2_2_	45.3 ± 11.8_2_	30.5 ± 8.0_1_	<0.001
Average Number of Patients per week	198.7 ± 273.2	74.0 ± 48.5_1_	37.9 ± 37.2_1_	503.8 ± 310.3_2_^a^	<0.001
	% (*n*)	% (*n*)	% (*n*)	% (*n*)	*p*-value
Gender (female)	69.8 (67)	69.4 (25)_a_	90.0 (27)_b_	50.0 (15)_a_	0.003
Years of practice					
0–5 years	25.0 (24)	11.1 (4)_a_	16.7 (5)_a_	50.0 (15)_b_	0.007
6–10 years	24.0 (23)	22.2 (8)_a_	26.7 (8)_a_	23.3 (7)_a_	
11–20 years	19.8 (19)	27.8 (10)_a_	13.3 (4)_a_	16.7 (5)_a_	
21–30 years	14.6 (14)	13.9 (5)_a_	23.3 (7)_a_	6.7 (2)_a_	
More than 30 years	16.7 (16)	25.0 (9)_a_	20.0 (6)_a_	3.3 (1)_b_	
State of practice					
New South Wales	83.3 (80)	83.3 (30) _a,b_	73.3 (22)_b_	93.3 (28)_a_	0.57
Victoria	8.3 (8)	8.3 (3)_a_	13.3 (4)_a_	3.3 (1)_a_	
ACT	1.0 (1)	0.00 (0)_a_	3.3 (1)_a_	0.00 (0)_a_	
QLD	2.1 (2)	2.8 (1)_a_	0.00 (0)_a_	3.3 (1)_a_	
SA	3.1 (3)	2.8 (1)_a_	6.7 (2)_a_	0.0 (0)_a_	
TAS	2.1 (2)	2.8 (1)_a_	3.3 (1)_a_	0.0 (0)_a_	
WA	0.00 (0)	0.00 (0)	0.00 (0)	0.0 (0)	
NT	0.00 (0)	0.00 (0)	0.00 (0)	0.0 (0)	
Geographical classification of practice^b^					
Metropolitan	73.4 (69)	76.5 (26)_a_	60.0 (18)_a_	83.3 (25)_a_	0.156
Regional	4.3 (4)	2.9 (1)_a_	3.3 (1)_a_	6.7 (2)_a_	
Rural/Remote	22.3 (21)	20.6 (7)_a,b_	36.7 (11)_b_	10.0 (3)_a_	
Employment status					
Full time	40.6 (39)	36.1 (13)_a_	46.7 (14)_a_	40.0 (12)_a_	0.003
Part time	45.8 (44)	63.9 (23)_a_	40.0 (12)_a,b_	30.0 (9)_b_	
Casual	13.5 (13)	0.0 (0)_a_	30.8 (4)_b_	69.2 (9)_b_	

**^a^** This is based on the average number of prescriptions dispensed per week in the pharmacy.

^b^ Categorization based on the modified Monash model whereby MM1  =  metropolitan, MM2 = regional and MM3 to MM7  =  rural and remote.

The numerical subscripts denote means for groups in homogenous subsets.

Percentage (%) is expressed as a proportion within each profession; where there is missing data, the denominator from each proportion calculated is also expressed.

The same alphabetical subscript indicates that column proportions do not differ significantly from each other.

Participants who were interviewed (*n*  =  45: 17 GPs, 14 community nurses, and 14 community pharmacists) were more likely to be female, *X*^2^ (1)  =  4.187, *p*  =  0.041. No further demographic differences were observed. With respect to attributes of consideration when implementing DHIs, participants who were interviewed rated data security (median  =  6.00, *U*  =  879.5, *p*  =  0.037, *r*  =  −0.021) and a trustworthy repository (median  =  6.00, *U*  =  888.0, *p*  =  0.039, *r*  =  −0.210) as more important compared to those who were not interviewed. Supplemental Appendix C provides a summary of comparisons between the two groups.

### Comparison of DHI use across healthcare professionals

Table 2 provides a summary of healthcare professionals' (HCPs) current use and attitudes towards DHIs. Of the three professions, GPs were more likely to report engaging with these technologies in general, *X*^2^(2)  =  11.336, *p*  =  0.003, as well as those specific for sleep health, *X*^2^(2)  =  15.318, *p* < 0.001. These findings also corresponded to the higher scores observed for GPs with respect to their perceived level of familiarity with sleep DHIs, *F*(2, 93)  =  4.905, *p* < 0.009. Post-hoc comparisons indicated significant mean differences between GPs and nurses only (*p*  =  0.007). From the perspective of DHI implementation in primary care, the needs, and priorities were comparable across the three professions. The main differences were observed for the attribute ‘DHI developed by health care professionals or clinical bodies’, which was rated as least important by pharmacists, *F*(2, 93)  =  6.077, *p*  =  0.003. Post-hoc comparisons indicated significant mean differences in ratings between pharmacists (*x̄*  =  4.8, SD  =  1.0) and nurses (*x̄*  =  5.5, SD  =  0.7) (*p*  =  0.002). Relatedly, pharmacists also reported the highest scores for ‘financial incentives for HCPs’, *F*(2, 92)  =  5.356, *p*  =  0.006. Post-hoc analysis only showed significant mean differences in ratings between pharmacists (*x̄*  =  3.8, SD  =  1.4) and GPs (*x̄*  =  2.4, SD  =  2.0) (*p*  =  0.005). Looking more closely at participants’ interest in sleep DHI functionality, educational features (83.3%) were the most frequently selected followed by behavioural management (79.2%), tracking (75.0%) and diagnostic and screening features (55.2%). Between disciplines, GPs endorsed a stronger level of interest in the diagnostic and screening features of a sleep DHI compared to nurses and pharmacists, *X*^2^(2)  =  9.394, *p*  =  0.009.

**Table 2.** Current use and attitudes towards sleep-related digital health by profession.

	Total sample (*n* = 96) mean ± SD	General practitioner (*n* = 36) mean ± SD	Community nurse (*n* = 30) mean ± SD	Community pharmacist (*n* = 30) mean ± SD	*p*-value
Digital health intervention (DHI) familiarity	1.7 ± 1.1	1.9 ± 1.0_1_	1.3 ± 1.0_1_	1.7 ± 1.14_1_	0.084
Sleep DHI familiarity	0.7 ± 0.9	1.0 ± 1.1_2_	0.4 ± 0.7_1_	0.7 ± 0.74_1,2_	0.009
Attributes when considering implementation of DHIs					
Data security	4.9 ± 1.4	4.9 ± 1.3_1_	4.9 ± 1.5_1_	5.0 ± 1.3_1_	0.903
Privacy policy	5.2 ± 1.2	5.2 ± 1.2_1_	5.2 ± 1.3_1_	5.2 ± 1.2 _1_	0.991
Legal compliance	5.2 ± 1.3	5.2 ± 1.1_1_	5.1 ± 1.5_1_	5.1 ± 1.3_1_	0.885
Trustworthy repository	5.2 ± 1.0	5.1 ± 0.8_1_	5.4 ± 1.1_1_	5.0 ± 0.8_1_	0.266
Endorsement	4.8 ± 1.1	4.8 ± 1.0_1_	5.1 ± 1.2_1_	4.5 ± 1.2_1_	0.153
DHI developed by healthcare professionals or clinical bodies	5.2 ± 0.9	5.2 ± 0.7_2_	5.5 ± 0.7_2_	4.8 ± 1.0_1_	0.003
Demonstrated efficacy	5.1 ± 0.9	4.9 ± 1.0_1_	5.2 ± 1.0_1_	5.1 ± 0.9_1_	0.384
Training	4.9 ± 1.0	4.7 ± 1.1_1_	5.2 ± 0.8_1_	4.9 ± 1.0_1_	0.159
Financial incentives for HCP^c^	3.1 ± 1.9	2.4 ± 2.0_1_	3.3 ± 1.9_1_	3.8 ± 1.4_2_	0.005
Cost to patient	4.8 ± 0.9	4.7 ± 1.0_1_	5.0 ± 1.0_1_	4.7 ± 0.9_1_	0.421
** **	**% (*n*)**	**% (*n*)**	**% (*n*)**	**% (*n*)**	***p*-value**
Sleep-related problems encountered in practice					
Nil	2.1 (2)	0.0 (0)_a_	3.3 (1)_a_	3.3 (1)_a_	0.059
1–5	44.8 (43)	50.0 (18)_a_	53.3 (16)_a_	30.0 (9)_a_	
6–10	30.2 (29)	33.3 (12)_a_	33.3 (10)_a_	23.3 (7)_a_	
11–15	13.5 (13)	11.1 (4)_a_	10.0 (3)_a_	20.0 (6)_a_	
16+	9.4 (9)	5.6 (2)_a_	0.0 (0)_a_	23.3 (7)_b_	
Use of DHI in practice (yes)	53.1 (51)	75.0 (27)_a_	43.3 (13)_b_	36.7 (11)_b_	0.003
Use of sleep DHI in practice (yes)	27.1 (26)	50.0 (18)_a_	13.3 (4)_b_	13.3 (4)_b_	<0.001
Interest in using Sleep DHI diagnostic (yes)	55.2 (53)	75.0 (27)_a_	46.7 (14)_b_	40.0 (12)_b_	0.009
Interest in using Sleep DHI tracking (yes)	75.0 (72)	80.6 (29)_a_	73.3 (22)_a_	70.0 (21)_a_	0.595
Interest in using Sleep DHI educational (yes)	83.3 (80)	94.4 (34)_a_	76.7 (23)_b_	76.7 (23)_b_	0.077
Interest in using Sleep DHI behavioural (yes)	79.2 (76)	88.9 (32)_a_	70.0 (21)_a_	76.7 (23)_a_	0.157
Interviewed (yes)	46.9 (45)	47.2 (17)_a_	46.7 (14)_a_	46.7 (14)_a_	0.999

The numerical subscripts denote means for groups in homogenous subsets.

Percentage (%) is expressed as a proportion within each profession; where there is missing data, the denominator from each proportion calculated is also expressed.

The same alphabetical subscript indicates that column proportions do not differ significantly from each other.

### Qualitative thematic synthesis

Analysis of the qualitative data revealed three key emergent themes: Scope for DHIs in current practice, Practice gaps and training needs, and Envisioning a model of care using sleep DHIs. Tables 3 to 5 provide a summary of the thematic synthesis and illustrative quotes for emergent themes.

**Table 3.** Theme 1: Scope for digital health interventions (DHIs) in current practice.

Subtheme	Summary	Illustrative quotes
1.1 Knowledge, beliefs, and experiences with DHIs	• Primary care providers describe a broad range of products identified as a DHI: app-based interventions, wearables and informatic systems. •DHIs are considered a ‘vague’ term encompassing the range of platforms and services. •Variability in experience with DHIs; those working in mental health are more likely to integrate app-based interventions in their practice.	• ‘Digital health interventions? I kind of think of things like online CBT … I guess more recently in particular, I guess in the current climate with everything becoming more-telehealth becoming a lot more common, obviously there's a lot more in terms of telehealth consultations, Skype consultations and so on…’. **(P18_GP: F; age: 36; practice: 11–20 years)** •‘Okay, so in terms of digital health interventions and everything, the term is actually pretty vague itself, but I would actually assume that even things like the dispensing software and everything that you can actually keep the history of what the patient is on, document any clinical notes that would be part of your digital health’. **(P14_Pharmacist: M; age: 27; practice: 0–5 years)** •‘Well, it's a wide variety really. We’re talking to people about using different apps. I mean, even the basis of sort of saying to someone, “Just stick an alarm on to remember to take your medication”. … We’re talking to people about what digital health is, [as it's] becoming more and more available. And at the moment, of course, telehealth is taking off…’. **(P04_Nurse: F; age: 60; practice: 30+ years)**
1.2 COVID-19: A catalyst for digitalisation in primary care practice	• Infrastructure upgrades and personnel upskilling in response to COVID-19 have facilitated the digitalisation of primary care. •COVID-19 has brought about new changes to practice (e.g. e-script and medicare item numbers). •Primary care providers realise new and expected ways to deliver healthcare to patients at their practice. •Shifting attitudes towards digital health platforms among primary care providers and patients – acceptance and positive health outcomes.	• ‘Lots of doctors have been advocating for it for many decades. So the fact that there is now telehealth item number and all those things has really revolutionized the general practice… As of the last three or four months, there's a new software which we’ve set up called e-Scripts …and I type in the patient's mobile and it sends from my software, as a QR code, directly to their mobile…’. **(P31_GP: F; age: 46; practice: 11–20 years).** •‘Also, we’ve had another patient who needed her wounds cleaned and we did video conferencing, so the daughter went to the mom's home with her laptop and the nurse was able to guide.The daughter was doing the wound care and she was guided by the nurse at the community health building, in the center, and she was able to do what she was doing and advise her what to do. And that worked very well and her wound healed’. **(P07_Nurse: F; age: 51; practice: 21–30 years)** •‘We are seeing more and more people coming in, you know, with just a QR code on their mobile phones, and then all we’ve got to do is just scan them and all the information is already there, and we pass them the prescription’. **(P26_Pharmacist: F; age: 27; practice: 0–5 years)**
1.3 Perceived need and place for DHIs	• Potential role of DHIs in improving patient access to treatment and can serve as an adjunct to face-to-face treatment. •Overcoming staff shortages in rural/remote settings and in aged-care. •Sleep DHIs provide an alternative option, potentially offsetting medication use in areas of high-volume prescribing such as aged-care. •Meeting post-pandemic demand for sleep/mental health services. •Concerns around the cumulative screen time when using DHI, which may worsen sleep.	• ‘I think so, yes. Sleep health is becoming a problem. And I’m not sure about it—I think even post-pandemic it's going to be an issue because once the pandemic is over, there's going to be a lot of depression and side effects of depression which includes insomnia and all these things’. **(P17_Pharmacist: M; age: 43; practice: 11–20 years)** •‘…and as far as helping them, you know, people sometimes want sleeping tablets. Really, it's I think often they want to sort out their problem, they don’t necessarily want sleeping tablets. But we don’t have that much to offer them’. **(P01_GP: F; age: 51; practice: 21–30 years)** •‘I see it increasing and I see that it (DHI) could have a really positive effect, and I think patients will be increasingly keen to engage…’. **(P18_GP: F; age: 36; practice: 11–20 years)** •‘So, I think, again, for the right person at the right time, for the right issue, there's a lot of strength in [the] availability [of DHIs] there…but the other place where I think it can be useful is also as an adjunct to actual face-to-face stuff. So, I think it’d be helpful both ways’. **(P19_Nurse: M; age: 38; practice: 11–20 years).** •‘So, if I have to keep my phone near me for tracking, I probably would be tempted to also look at Facebook and Instagram and other things before I’m sleeping. So I think it would inadvertently affect my sleep anyway’. **(P20_Pharmacist: F; age: 27; practice: 0–5 years)**

**Table 4.** Theme 2: Practice Gaps and Training Needs.

Subtheme	Summary	Illustrative quotes
2.1 Knowledge about sleep health: Breadth versus depth	• Sleep complaints are common in primary care – insomnia and obstructive sleep apnea most prevalent. •Perceived current knowledge of sleep physiology and management options to lack depth. •Sleep health knowledge and training variable across professions; dependent on self-interest/professional context in selecting CPD activities. •Competing demands with other therapeutic areas.	• ‘…as a pharmacist I feel like we know a bit of everything, but we are not like—we don’t know in-depth for every topic’. **(P02_Pharmacist: F; age: 28; practice: 6–10 years)** •‘…we do have a sleep questionnaire but it's ‘Are you sleeping at night? Yes or No?’ And if there's a no, then there's no really what's keeping them awake and what pain relief are they taking. It doesn’t guide you to another source of health’. **(P7_Nurse: F; age: 51; practice: 21–30 years)** •‘I think it becomes very specialized for sleep. So those who are working there in the sleep unit, I think apart from that, nobody else will know a lot’. **(P43_Nurse: F; age: 50; practice: 21–30 years)** •‘I generally had to access it myself over the years. So there is available—it's not part of your standard training, but there is available if you realize there's a gap in your education and you seek to fill that gap’. **(P42_GP: F; age: 60; practice: 30+ years)** •‘The various things that I have picked up over the years have come from mental health seminars, particularly addressing treatment of anxiety and depression where sleep disruption has been covered and sleep hygiene has been covered as part of that mental health training’. **(P16_GP: F; age: 62; practice: 30+ years)**
2.2 Expectations for a sleep digital health intervention (DHI)	• Participants drew on current digital health experiences with informatic systems, apps, and wearable devices. •Focus on sleep tracking and education features; integration with technological affordances (e.g. notification/pop-ups) to reinforce positive sleep behaviour changes. •Focus needs to be directed towards tailoring DHI to individual needs rather than a ‘one-size-fits-all’ approach. •Communication interface within the app to enable different healthcare professionals to be informed about the patients’ condition.	• ‘…well, things to help set up good sleep hygiene, so an alarm to say, ‘Now is the time to switch off the television or switch off the computer or start relaxing,’ and then another alarm saying, ‘This is the time to actually go to bed’. **(P16_GP: F; age: 62; practice: 30+ years)** •‘I’m thinking similar to what the Fitbit is, and basically it syncs with the phone … It sort of structures your day, that this is when you should sleep, this is when you should wake up and—I don’t know how it's going to work, but some sort of positive reward system…like when you wake up it plays your favorite song’. **(P17_Pharmacist: M; age: 43; practice: 11–20 years)** •‘It must be very user-friendly, it mustn’t have too many hurdles to jump through every time you open it up or have to get onto something’. **(P30_GP: F; age: 58; practice: 30+ years)** •‘Might even be worthwhile considering having a capacity to incorporate wearable technology like your Apple watch or your Garmin into this, so you know how much they’re actually sleeping or what they’ve been recording on it. Maybe probably including who are the clinicians in their care team so that they know who they can go to for help or who they have been going to for help. Some goal setting in that thing, so what their goals are with their sleep patterns and what strategies they’re currently trialling so that they have a clear plan in their mind’. **(P45_Nurse: F; age: 36; practice: 0–5 years)**
2.3 Readiness to implement sleep DHIs	• From a technological infrastructure perspective, participants felt their practices were well-equipped digitally. •DHIs perceived as an extension of their practice but felt the need to gain a greater depth of knowledge about sleep health and management approaches specifically. •Considerations for patient uptake and support needed for onboarding (e.g. demonstration devices). •Primary care providers/staff pre-trialling product prior to recommending to patients.	• ‘You know, like, as long as I understand how it works…Once I understand how it works, it would be quite easy for me to tell them’. **(P02_Pharmacist: F; age: 28; Practice: 6–10 years)** •‘And most of the time our guys will trial it themselves just because they need to know—if they’re going to recommend it, they need to know how to use it and all the recommendations need to be evidence-based’. **(P3_Pharmacist: F; Age: 37; Practice:11–20 years)** •‘If I knew about the app and had a little peruse of it myself and felt confident to be able to talk about the benefits of the app and how it worked and some troubleshooting, I think I’d be pretty confident to be able to help the patient’. **(P37_Nurse: F; age: 42; practice: 21–30 years)** •‘Well, I wouldn’t be hugely confident. I think the only one I do—I do often recommend like a meditation app or like a sleep kind of program that might—that's what I liked about Calm, because it does have a bit of an education link to it as well. But if it was things like something that might give us more information about—a bit like a sleep study, I suppose, then I think I’d need more education on that kind of stuff. Yeah’. **(P41_GP: F; age: 41; practice: 11–20 yerrs)**

**Table 5.** Theme 3 envisioning a model of care using sleep digital health interventions (DHIs).

Subtheme	Summary	Illustrative quotes
3.1 Accessing sleep DHIs in primary care: A vision for future practice	• Primary care providers unanimously agreed medical supervision would be required to guide patients and avoid sleep DHI from becoming a form of self-treatment. •GPs and nurses saw a limited role for pharmacists in implementing DHIs – noting their expertise is solely in pharmacotherapy. •Conceptualised different pathways of patient access to sleep DHI; balancing between access versus risk of self-treatment. •Ideas ranged from adopting a prescription-based system to a tiered-access system based on consulting with a primary care provider.	• ‘I would like to think that GP is the right person to do that because they’re well aware, hopefully, as the gatekeeper to medical and healthcare, and hopefully they know the patient well and they know all about their health rather than just about a little bit… So, the pharmacist might have some information about it, but not be the one who would actually make the decision to prescribe it’. **(P16_GP: F; age: 62: practice: 30+ years)** •‘So anyone can get that app, but they can only assess certain information …in order to access more deeper information, you need maybe like a call from the doctor or the pharmacy because otherwise you’ll be self-diagnosing…’. **(P02_Pharmacist: F; age: 28; practice: 6–10 years)** •‘I suppose if their (pharmacist) realm is pharmacological stuff—I don’t know that it necessarily falls within their scope, but certainly GPs, nurses, psychologists, psychiatrists, I think it would be useful and reasonable for it to be recommended by those individuals’. **(P19_Nurse: M; age: 38; practice: 11–20 years)**
3.2 Practice readiness for implementing sleep DHIs: Perceived barriers and enablers	• Despite infrastructure upgrades and staff upskilling, investment in human resources was identified as important for implementing DHIs in practice. •Willingness to invest in personnel contingent on patient demand at the respective practices to ensure it is a financially sustainable service. •Clearer reimbursement structures are needed for the delivery of sleep DHIs, especially for pharmacists who are traditionally paid for dispensing medicines. •The commercial space of community pharmacy poses additional challenges such as alignment with the company strategy for those operating in banner groups or store-level allocation of space for services versus merchandise.	• ‘Whenever you’re teaching someone anything, you need time because I’m going to have questions. So it's not going to be a simple—for someone it might be a simple 2-min job or I’ll download the app and they run it already and they click and a few things. While then you might have someone that they’re going to need a bit more education regarding even downloading the app or, you know, registering for the app. You know, that's going to take a lot more time and that's the main factor’. **(P05_Nurse: F; age:33: practice: 6–10 years)** •‘I think financially is the major thing. My practice would consider it if they’d make money off it’. **(P04_Nurse: F; age: 60; practice: 30+ years)** •‘Problem about investment, you know, how much time, how much dollars, and especially if you don’t even know if it's going to work. So, I would have to know that it's hugely endorsed, that it's beneficial before I was to [invest]’. **(P30_GP: F; age: 58; practice: 30+ years)** •‘I would say in terms of staffing, yes. Cost, not so much because if it's just an app, probably people would just get it off the app store and everything…’. **(P14_Pharmacist: M; age: 27; practice: 0–5 years)** •‘A lot of pharmacy KPIs are based on things like claiming meds checks and claiming all kinds of things from the Seventh Community Pharmacy Agreement. So this could be run as part of it, part of the health services that are part of the agreement maybe, but it just depends on how much is in the government's budget as well to release for these programs’. **(P21_Pharmacist: F; age: 31; practice: 6–10 years)** •‘It’d have to be pretty low because I don’t have space—I need a refit in the current pharmacy. I’ve got $10,000 or more worth of sleep apnea stuff that really should be out on shelves to be sold, that I can’t put on shelves’. **(P34_Pharmacist: M; age: 38; practice: 11–20 years)**
3.3. Government, regulations and policymaking for DHIs	• The range of app-based products for ‘health’ makes it challenging for clinicians to make recommendations. •Recommending non-evidence-based DHIs can potentially undermine public trust in the respective professions. •Need for stronger regulatory oversight in providing therapeutic standards and establishing an evidence base for DHIs that healthcare professionals can reliably refer to. •Privacy issues were not a concern for participants. Perceived individuals who engage with DHIs are self-selected and are comfortable with their current level of use/disclosure of personal information.	• ‘… regulations must be there. We must not go forward without having clear rules and structure because otherwise we’ll end up with a diluted quality and an unreliable product, and that will affect [patient] trust’. **(P27_GP: M; age: 67; practice: 30+ years)** •‘I guess I wouldn’t be, because I don’t know of any current apps—and then even if I did, I don’t know if they’ve got scientific backing and stuff, so I don’t know enough about it to recommend one’. **(P33_GP: F; age: 36; practice: 6–10 years)** •‘I think it's a bit of a minefield at the moment. There are so many available. It's a bit like Dr Google, you know. How does someone know what is the best and what is the most appropriate intervention for them if there are no guidelines or if there's no evidence to support that particular intervention?’ **(P11_Nurse: F; age: 50; practice: 0–5 years)** •‘…if I’m just recommending a random app and the next day there's a data breach or it's not accurate at all or something happens to the app, it would come back to us that we haven’t been recommending the correct app or like a neglect…’. **(P02_Pharmacist: F; age: 28; practice: 6–10 years)** •‘I think the people that I see, who have been using them, have obviously worked out that they’re quite comfortable with the use and that's why they’re using them’. **(P16_GP: F; age: 62; practice: 30+ years)** •‘Look, the ones who are tech-savvy tend to be very aware of their privacy level. You know, they know how to use their devices well’. **(P23_Nurse: F; age: 59; practice: 30+ years)** •‘The objections I’ve had to suggestions of like an app or digital health intervention has just been namely around just generic “I’m not interested” or “I’m not very good with technology” sort of things. But nobody specifically mentioned anything privacy related’. **(P19_Nurse: M: age: 38; practice: 11–20 years)**

### Theme 1: Scope for DHIs in current practice

#### Knowledge, beliefs, and experiences with DHIs

Among participants, the term DHIs was described as a ‘vague’ umbrella term that involved the use of technology to improve health delivery/patient outcomes. Key products mentioned in the interviews included smart phone applications, health informatics systems (e.g. prescribing systems, dispensing systems, and My Health Records), telehealth consultations, online self-guided programs, websites, as well as tracking devices. Participants’ understanding of DHIs stemmed from their own experiences either through engaging with technology at their workplace or through personal experiences with using tracking devices (e.g. Apple Watch) and smart phone applications. However, participants’ familiarity and implementation of DHIs varied according to their practice specialities. Those working closely in a mental health setting were more likely to integrate relaxation and mindfulness smartphone applications (e.g. Headspace) as part of standard patient care. While 26.8% of the total survey sample endorsed the use of sleep DHIs at their practice, open-text responses and the qualitative data suggest that current sleep DHI use mostly revolves around accessing online sleep health information and providing information printouts for interested patients.

#### COVID-19: A catalyst for digitalisation in primary care practice

Across the three professions, there was an apparent positive shift in attitudes towards digital health due to the rapid digitalisation of the workplace (i.e. telehealth and e-scripts), upskilling, workflow adjustments and upgraded infrastructure that is ‘already all set up’ (P8_GP: F; age: 66; practice: 30+ years) in response to COVID-19. Contextually, the pandemic has brought about important disruptions to practice, catalysing a shift in attitudes among policy makers such as the introduction of a medicare item for telehealth. In such a practice context, participants uncovered new and unexpected ways to deliver healthcare to patients. Patients were perceived to be more accepting and willing to use digital health technologies such as telehealth consultations and e-scripts. In the case of older patients at the respective practices, many were able to engage with digital health, albeit through the support of family members, to achieve positive outcomes.

#### Perceived need and place for DHIs

Participants at large spoke about the potential utility of DHIs to improve the efficiency of health services delivery. Specifically, DHIs could potentially overcome staff shortages in aged-care and in rural/remote settings where retaining expertise proved challenging. The utility of DHIs was seen as most applicable in the areas of health tracking (e.g. *step count*) and mental health drawing on explicit proprietary examples such as Calm and Headspace. Specifically, the development in sleep health DHIs was perceived to be contextually timely with the observed impact of COVID-19 on sleep and mental health in the broader community. Within aged-care, sleep DHIs could potentially circumvent the issue of relying on pharmacotherapy due to a lack of available treatment options. Nonetheless, a small subset of participants did express concerns about using DHIs to address sleep complaints. Participants recognised the paradox of addressing sleep complaints with a device that can potentially impact sleep given its use in proximity to bedtimes, along with culminative screen time that people are exposed to in their daily lives.

### Theme 2: Practice gaps and training needs

#### Knowledge about sleep health: Breadth versus depth

In general, participants described the limited coverage of sleep health as part of their training at university or existing continuing professional development (CPD) activities. Differences in training/knowledge of sleep health and sleep disorders between HCPs were attributed to working in different speciality practice contexts such as a sleep laboratory, pharmacy providing CPAP services, or clinical settings with a mental health focus. Except for three nurses (P23, P24, and P25) who had received formal CBT-I training, sleep hygiene and lifestyle factors were the predominant non-pharmacological treatment options offered to patients across the three professions. Participants described their knowledge of sleep health as ‘knowing a little about everything’ (P03_Pharmacist: F; age: 37; practice: 11–20 years), identifying gaps in their understanding of the underlying pathophysiology of sleep disorders, diagnosis, treatment options, and referral pathways. A subset of participants further highlighted limitations of existing tools for sleep health which provide prompts to assess sleep health but ‘…doesn’t guide you to another source of health’ (P07_Nurse: F; age: 51; practice: 21–30 years) for patients who need further care. Importantly, across the three professions, there was an interest in upskilling therapeutic knowledge in sleep with suggestions for increasing training as part of their CPD (i.e. online modules, webinars, or workshops) or as part of workplace meetings. However, training affordability and competing demands between different therapeutic areas remain important barriers to HCP engagement.

#### Expectations for a sleep DHI

Descriptions capturing what a sleep DHI would entail were closely related to participants’ current knowledge about sleep health and drew on the platforms and devices they were already familiar with (e.g. Fitbit tracking systems and calm). Key areas of focus for a sleep DHI included sleep tracking (e.g. total sleep hours and sleep stages), education modules and capitalising on the technological capabilities to engage the user and facilitate sleep behaviour changes. Suggestions included the use of algorithms which strategically schedule pop-up alerts based on sleep patterns such as ‘stop screen time now’ (P33_GP: F; age: 36; practice: 6-10 years) or ‘waking up to a favourite song’ (P17_Pharmacist: M; age: 43; practice: 11–20 yrs) as positive behaviour reinforcement for regular sleep–wake schedules. Tailoring the DHI to the individual was highlighted as an important feature in the development of sleep DHIs rather than a ‘one-size-fits-all’ (P16_GP: F; age: 62; practice: 30+ years). A pharmacist drew parallels to the limitations of existing digital health products which are ‘very general and don’t speak to most people in specific…’ (P21_Pharmacist: F; age: 31; practice: 6–10 years). Closely related was the need for an intuitive user-interface, which will facilitate patient onboarding and the clinicians’ ability to access and use data to inform patient care.

#### Readiness to implement sleep DHIs

With respect to the confidence and feasibility of embedding sleep DHIs into practice, participants described their readiness in terms of two separate domains. From the perspective of technological infrastructure (e.g. computer hardware), participants felt ‘most of the barriers have been overcome’ (P08_GP: F; age: 66, practice: 30+  years), considering the recent upgrades and upskilling at the respective practices in response to COVID-19. As such, implementing new digital health technologies was perceived as an extension of their current practice. In contrast, participants were less confident in their depth of knowledge concerning the physiological mechanisms underlying sleep disorders and their management. Another factor central to the implementation of sleep DHIs was patient uptake. Participants highlighted the importance of test-driving digital health products to gauge usability and identify potential pitfalls to facilitate patient education and counselling. Specifically, having demonstration devices, user guides and patient-centred education materials that are written in a way that ‘consumers will find useful’ (P19_Nurse: M; age: 38; practice: 11–20 years) would enhance the patient onboarding process.

### Theme 3: Envisioning a model of care using sleep DHIs

#### Accessing sleep DHIs in primary care: A vision for future practice

Participants from the respective professional groups articulated different ideas for the pathway to allow patients in the community to access sleep DHIs ranging from ‘anyone should be able to access it’ (P18_GP: F; age: 36; practice: 11–20 years) through to needing a prescription. Despite the range of proposed access options, the involvement and oversight by an HCP were deemed necessary by all three professions. GPs and community nurses generally thought sleep DHIs are best prescribed to patients, drawing parallels to ‘the same way people get prescriptions for knee-strengthening exercises for osteoarthritis’ (P16_GP: F; age: 62; practice: 30+ years). Nurses working in a practice team with other GPs would ‘potentially okay it with the doctor’ (P06_Nurse: F; age: 30; practice: 6–10 years). Both GPs and nurses believed pharmacists would adopt a support role for implementing sleep DHIs, noting that pharmacists’ expertise was largely in medicines. However, all three professions agreed that direct product requests for sleep aids were a unique opportunity for pharmacists to ‘flag patients’ (P31_GP: F; age: 36; practice: 11–20 years) to educate them about sleep DHIs. Extending from this concept, pharmacists further suggested a stepped approach to accessing the sleep DHI, which can operate as a ‘two-tiered sort of program’ (P21_ Pharmacist: F; age: 31; practice: 6–10 years), offering patient access to general education modules while advanced functions/treatment modules would only be unlocked upon consultation with an HCP.

#### Practice readiness for implementing sleep DHIs: Perceived barriers and enablers

Participants felt ready to implement DHIs in general given the technological infrastructure upgrades and staff upskilling at the respective practices in response to COVID-19. However, limited time and anticipated workload increases were identified as key barriers to implementing DHIs that specifically targeted sleep. There was recognition that investing in more staff would be necessary to build capacity for delivering these services. Willingness to invest in human resources would be contingent on the financial sustainability of the DHIs as ‘it has to run as a profitable practice’ (P23_Nurse: F; age: 59; practice: 30+ years). Unlike technologies that are systemically built for the broader clientele base (e.g. telehealth consultations and e-scripts), participants’ responses implicitly categorised sleep DHIs as a speciality service, which may not have the same level of patient demand. Relatedly, HCP reimbursement was another area of concern. While GPs and nurses commented on the need to identify an appropriate time allocation for DHI implementation, community pharmacists identified a broader range of issues that need to be addressed. Firstly, pharmacists needed more clarity on how DHIs would fit within the Community Pharmacy Agreements (CPAs)^1^ as DHIs represent a significant departure from their current provision of medicines.

#### Government, regulations, and policymaking for DHIs

Currently, participants felt overwhelmed by the sheer number of health apps/devices on the market, which lack information on safety and efficacy, noting that there is ‘so much quackery out there’ (P30_GP: F; age: 58; practice: 30+ years). From a medicolegal perspective, participants highlighted the need for better government involvement in providing therapeutic standards and greater regulatory oversight before digital health products come to market, as per medical devices and medications. Such an approach would enhance the consistency of DHI use while instilling confidence in the general public to use DHIs as part of their care. Importantly, proven clinical efficacy and safety would ‘take away the challenges’ (P23, Nurse: F; age: 59; practice: 30+ years) for clinicians to embed DHIs in their routine practice, allaying concerns around issues of professional liability and compromised public trust in the respective professions. In contrast, participants did not perceive data privacy and security issues as being a huge concern for patients at the respective practice. Many described DHI users as self-selected individuals who ‘worked out they were quite comfortable with their use’ (P16_GP: F; age: 62; practice: 30+ years) of such products. This was largely attributed to the expansive ways in which smartphones and apps are used day to day where there is ‘…no difference…’ (P03_ Pharmacist: F; age: 37; practice: 11–20 years) when it comes to using DHIs. Participants also highlighted patient trust in the HCP as key to allaying patient concerns about the legitimacy of the DHI and data security. As such, transparency about the evidence supporting DHIs, the accreditation process, the types of information collected, and how and where this information is stored would be important to allow HCPs to help patients make informed choices.

## Discussion

To our knowledge, this is one of the first studies which has explored and triangulated the perspectives of primary care health professionals on the implementation and usage of sleep DHIs. Three key themes were identified following interviews with GPs, nurses, and community pharmacists: Scope for DHIs in current practice, Practice gaps and training needs*,* and Envisioning a model of care using Sleep DHIs. The data shows several gaps in the ability of health professionals to implement DHI use in primary care. This concern is predominantly related to clinical care pathways and whether the regulatory and reimbursement processes can be integrated into current clinical practice.

The response to COVID-19 has shifted primary care providers’ perspectives on digital health technologies and perceived an increase in patients’ acceptability and engagement with digital health platforms. However, participant interviews across the three professions confirmed the need for greater clarity for delineating digital health care pathways, patient access points, and integration with existing health informatic systems (e.g. prescribing and dispensing software). The latter is a well-established clinician-level barrier to the uptake of DHIs,^37^ which is increasingly important given the proliferation of technology-enabled care across disease states, including sleep health.^38^ As such, multi-stakeholder consultation in co-designing the informatic system interface is needed for aligning patient digital health needs with clinician workflow.^39,40^ One strategy is to capitalise on the existing system structures to lower the threshold of practice uptake. Internationally, DHI developers are increasingly embedding digital therapeutics through a pharmacy pathway, to capitalise on the existing informatics systems that connect patient records with insurers.^41^ Another point of integration could be the expansion of the prescriber formulary to include DHIs as demonstrated by Byambasuren et al.^42^ There is also scope for expanding the healthcare team to include a digital navigator with expertise in digital health who can facilitate the implementation of new technologies in practice.^43^

Differences between the professions were also observed. Survey responses showed that GPs were most familiar with sleep DHIs and were more likely, of the three professions, to use general DHIs and sleep-specific DHIs in their current practice. From Table 1, nurses and pharmacists most frequently reported being employed on a casual status, which may limit opportunities for scaling up DHIs as they are often employed on a needs basis to address staffing shortfalls. Differences in reimbursement needs were also apparent across the three professions. While GPs and nurses sought clarity in terms of how sleep DHIs would fit into the existing consultation structures, pharmacists identified more barriers, endorsing stronger importance of financial incentives (*p*  =  0.006) compared to GPs and nurses (Table 2). Current reimbursement models for community pharmacies are that they operate in a retail environment and largely focus on the provision of medicines many of which are subsidized by the government through the Pharmaceutical Benefits Scheme in Australia.^44,45^ This may explain why both GPs and nurses in the study perceived a limited role in pharmacist involvement in deploying sleep DHIs. Yet the National Academies of Sciences, Engineering, and Medicine Committee has recommended calling for a shift from fee-for-service models to hybrid reimbursement models involving healthcare teams rather than sole-providers.^46^ Interprofessional sleep health education opportunities represent an important area of development, as participants across professions were open to expanding their knowledge of sleep physiology and management.

In implementing sleep DHIs in practice, participants’ main concerns stemmed from the lack of regulation for the safety, quality, and efficacy of existing e-health products. Many commented on feeling overwhelmed by the sheer volume of products, making the selection of DHIs challenging in practice. These sentiments are reflected in participants’ survey responses highlighting the prioritisation of privacy policy, legal compliance, and having a trustworthy repository when considering or implementing DHIs (Table 2).^47,48^ In fact, clinician uncertainty often prevents the uptake of new interventions in practice changes,^49^ which may underpin the challenge of implementing sleep DHIs in primary care. Studies conducted in other therapeutic areas also highlight similar barriers to the uptake of DHIs in cardiovascular^50^ and diabetes care.^51,52^ Strengthening regulatory processes with evidence-based components relating to safety, efficacy, and quality is an important priority for expanding DHIs into routine care including sleep health. A key challenge for regulating the digital health marketplace is its rapid pace and evolution, posing difficulties for regulatory bodies to keep up with the changes/updates.^53^ Rodriguez-Villa and Torous^54^ proposed a public-interactive approach, which encourages individuals with real-world experiences, to comment on developers’ answers to a set of self-certification check-list questions. Currently, the US Food and Drug Administration (FDA) is piloting the ‘FDA Digital Health Software Pre-certification (Pre-Cert) Program’.^55^ While the program aimed to expedite the approval process with companies/enterprises with an existing track-record, it does provide a framework for identifying real-world effectiveness and potentially understanding important variations which arise between research and real-world settings.^56^

### Strengths and limitations

The strength of the current study stems from the mixed methods study design, corroborating qualitative and quantitative findings. This method increases an understanding of the complexities of implementing DHI, overcoming the methodological limitations of either method alone.^57^ Our sample size (*n*  =  97 for the online survey, *n*  =  45 for interviews) and inclusion of major professional stakeholders (i.e. GPs, community nurses, and community pharmacists) provides a comprehensive snapshot of Australian primary care. Importantly, recruitment coincided with the rapid workplace digitalisation in response to the COVID-19 pandemic, unveiling the most salient issues facing Australian primary care when integrating DHIs.

However, there are some limitations to consider. Our participants were a self-selected sample of primary care practitioners who are potentially more interested in digital health technologies compared to the general population. Nonetheless, these participants are more likely to be early adopters of DHI technologies, making their perspectives and experiences highly valuable for informing the early stages of piloting and implementation of DHIs. Another limitation of the current study was hypothetical bias. As participants were not shown an actual sleep DHI that they would implement, participants may overestimate the amount of time/money they would be willing to invest in expanding this area of practice/suitability of the DHI to their current practice may differ.^58^ Furthermore, the findings captured only provide a cross-sectional perspective of DHI use and integration at the height of the pandemic and participants’ perspectives may have changed, warranting the need for ongoing research to explore HCP needs and practice trends. Additionally, the instrument for capturing attributes for DHI implementation was not a validated measure. The median scores for the individual item suggest participants endorsed a high level of importance across a range of attributes. However, these findings need to be interpreted with caution as they are based on a single item. Furthermore, it is unclear how different cut-off scores would correlate or predict DHI uptake, emphasising the need for further validation for use in primary care.

## Conclusion

Sleep-related DHIs represent a promising avenue for primary care practice to meet patient demands for therapeutic sleep health treatments. While the disruption of COVID-19 provided a unique opportunity (i.e. personnel upskilling and infrastructure upgrades) to embed digital health solutions into routine practice, the interviews highlighted the uncertainties among primary care providers. These uncertainties stem from reimbursement concerns, training, as well as their practice environment. From a disciplinary perspective, there is a need to promote and expand the roles of different HCPs. Developing an operational framework for how DHIs are regulated and fit within current pathways of healthcare in Australia is extremely important. A key agenda in health policy is to allow DHIs to become part of routine care to improve patient outcomes with current finite health resources.

## Supplemental Material

sj-docx-1-dhj-10.1177_20552076231180970 - Supplemental material for Embedding digital sleep health into primary care practice: A triangulation of perspectives from general practitioners, nurses, and pharmacists Click here for additional data file. Supplemental material, sj-docx-1-dhj-10.1177_20552076231180970 for Embedding digital sleep health into primary care practice: A triangulation of perspectives from general practitioners, nurses, and pharmacists by Janet MY Cheung, Zoe Menczel Schrire, Melissa Aji, Matthew Rahimi, Helena Salomon, Iliana Doggett, Nicholas Glozier, Delwyn J. Bartlett, Keith Wong, Ronald R. Grunstein and Christopher J. Gordon in DIGITAL HEALTH

sj-docx-2-dhj-10.1177_20552076231180970 - Supplemental material for Embedding digital sleep health into primary care practice: A triangulation of perspectives from general practitioners, nurses, and pharmacists Click here for additional data file. Supplemental material, sj-docx-2-dhj-10.1177_20552076231180970 for Embedding digital sleep health into primary care practice: A triangulation of perspectives from general practitioners, nurses, and pharmacists by Janet MY Cheung, Zoe Menczel Schrire, Melissa Aji, Matthew Rahimi, Helena Salomon, Iliana Doggett, Nicholas Glozier, Delwyn J. Bartlett, Keith Wong, Ronald R. Grunstein and Christopher J. Gordon in DIGITAL HEALTH

sj-docx-3-dhj-10.1177_20552076231180970 - Supplemental material for Embedding digital sleep health into primary care practice: A triangulation of perspectives from general practitioners, nurses, and pharmacists Click here for additional data file. Supplemental material, sj-docx-3-dhj-10.1177_20552076231180970 for Embedding digital sleep health into primary care practice: A triangulation of perspectives from general practitioners, nurses, and pharmacists by Janet MY Cheung, Zoe Menczel Schrire, Melissa Aji, Matthew Rahimi, Helena Salomon, Iliana Doggett, Nicholas Glozier, Delwyn J. Bartlett, Keith Wong, Ronald R. Grunstein and Christopher J. Gordon in DIGITAL HEALTH

## Notes

1. CPAs outlines how the government reimburses community pharmacies for their professional services https://www1.health.gov.au/internet/main/publishing.nsf/Content/New-7th-Community-Pharmacy-Agreement.
