# Supplementary material for: Embedding digital sleep health into primary care practice: A triangulation of perspectives from general practitioners, nurses, and pharmacists
Source: Digit Health. 2023 Jun 23;9:20552076231180970. doi: 10.1177/20552076231180970 (PMC10291541; doi:10.1177/20552076231180970)
Supplement: sj-docx-1-dhj-10.1177_20552076231180970 - Supplemental material for Embedding digital sleep health into primary care practice: A triangulation of perspectives from general practitioners, nurses, and pharmacists [file sj-docx-1-dhj-10.1177_20552076231180970.docx]

**Appendix A. Core Attributes of Consideration when Implementing DHIs into Practice^[[1]](#footnote-1)^**

**Please rate the level importance of each attribute when considering implementing digital health interventions into your clinical practice:**

|  | **Not at all important** | **Low importance** | **Slightly important** | **Neutral** | **Moderately important** | **Very important** | **Extremely important** |
| --- | --- | --- | --- | --- | --- | --- | --- |
| 1. Level of data security   *Regulation of how collected data are stored, used and shared; use of a secure online server* | ⃝ | ⃝ | ⃝ | ⃝ | ⃝ | ⃝ | ⃝ |
| 1. Privacy policy   *Policy to maintain confidentiality, integrity and availability of user data* | ⃝ | ⃝ | ⃝ | ⃝ | ⃝ | ⃝ | ⃝ |
| 1. Legal compliance and regulation   *Therapeutic Goods Association (TGA) or other centralised regulation of digital health interventions* | ⃝ | ⃝ | ⃝ | ⃝ | ⃝ | ⃝ | ⃝ |
| 1. Trustworthy repository of evidence-based digital health interventions   *A centralised source to access digital health interventions vetted by a regulatory authority* | ⃝ | ⃝ | ⃝ | ⃝ | ⃝ | ⃝ | ⃝ |
| 1. Endorsement from notified and credible bodies   *Stamp of approval or endorsement from trusted sources such as government agencies, TGA, or RACGP* | ⃝ | ⃝ | ⃝ | ⃝ | ⃝ | ⃝ | ⃝ |
| 1. Development by health professionals or clinical bodies   *The development of the digital health intervention has been guided or contributed to by clinical experts, health care providers or clinical bodies* | ⃝ | ⃝ | ⃝ | ⃝ | ⃝ | ⃝ | ⃝ |
| 1. Published studies to demonstrate efficacy or safety   *The level of scientific evidence to support the digital health intervention as a therapeutic alternative* | ⃝ | ⃝ | ⃝ | ⃝ | ⃝ | ⃝ | ⃝ |
| 1. Health care professional education and training about the digital health intervention   *Access to educational and training materials for health care professionals on the specific functions, benefits and requirements of each digital health intervention* | ⃝ | ⃝ | ⃝ | ⃝ | ⃝ | ⃝ | ⃝ |
| 1. Financial incentives   *Financial mechanisms to encourage and support the organization or clinician prescribing digital health interventions* | ⃝ | ⃝ | ⃝ | ⃝ | ⃝ | ⃝ | ⃝ |
| 1. Cost to the patient   *The cost to the patient to utilise the digital health intervention* | ⃝ | ⃝ | ⃝ | ⃝ | ⃝ | ⃝ | ⃝ |

1. IItems adapted from the original questionnaire developed by Leigh and Ashall-Payne ^23^ to capture the importance of core attributes for participants to consider when implementing DHIs in practice. [↑](#footnote-ref-1)
