# Supplementary material for: Embedding digital sleep health into primary care practice: A triangulation of perspectives from general practitioners, nurses, and pharmacists
Source: Digit Health. 2023 Jun 23;9:20552076231180970. doi: 10.1177/20552076231180970 (PMC10291541; doi:10.1177/20552076231180970)
Supplement: sj-docx-2-dhj-10.1177_20552076231180970 - Supplemental material for Embedding digital sleep health into primary care practice: A triangulation of perspectives from general practitioners, nurses, and pharmacists [file sj-docx-2-dhj-10.1177_20552076231180970.docx]

**Appendix B. Interview guide**

| **Questions** | | **Prompts** | **Rationale** |
| --- | --- | --- | --- |
|  | **What has been your experiences when addressing sleep problems at your current practice?** | - Types of cases: severity; acute vs. chronic, comorbidities. - Number and type of scripts prescribed/ dispensed - Frequency of cases in practice - E.g. Insomnia, RLS, OSA | Establish context of practice with respect to the types of sleep problems and management approaches. |
|  | **What is your general approach for managing those sleep problems?** | - Pharmacological vs. non-pharmacological - CPAP - Lifestyle changes (e.g. exercise, reduce caffeine) - Perceived training needs in sleep health and limitations for current practice. - Specialist referral | Gauge knowledge of treatment options and referral pathways across HCPs |
|  | **What is your current experience with digital health interventions in your practice?** | - Understanding of the term ‘digital health interventions’ - Perceived usefulness of digital health interventions/technologies in achieving therapeutic outcomes - Perceived/actual issues with data security and privacy   **Experienced**:   - Technologies that you are familiar with (e.g., measurement tools, health apps or internet programs, telehealth programs, preventative health, electronic medical records) - What do you look for in a digital health technology? - Existing programs that are already embedded in your practice?   **If no experience**:   - Why haven’t you used digital health technologies previously? - Personal experience with digital health through health apps and/or wearable devices - How do you use the data captured through these devices? - Specific disease management that you think would benefit most through digital health? | Gauge practitioner beliefs and attitudes towards digital health interventions and technologies in general. |
|  | **What is your impression of the level of interest/acceptability of technologies among your patients?**  **What about for these technologies for your patients sleep problems?** | - Demographic of clientele (health literacy, age, affluence, etc) - General impression: positive vs. negative - Patient use of wearables e.g. Apple watch, Fitbit, etc. - Have patients discussed health apps with you? | Practitioner perception of their patients.  **Hypothesis**: this might be an important implicit barrier/enabler to the implementation of digital health technology as it determines if there is a market. |
|  | **Would you consider using digital health technologies as a mainstream therapeutic like a prescribed medication for sleep problems?** | - Do you think digital health interventions need to be regulated like a medicine?   - Equivalent to the Therapeutic Goods Administration framework: safety, quality and efficacy. - Published studies demonstrating efficacy? - Government/Professional bodies regulating/accrediting digital health interventions?   - Pharmacist = Pharmaceutical Society of Australia   - GP= Medical Society of Australia   - Nurse= Australian Primary Health Care Nurses Association - Placing digital technologies as a therapeutic on the Pharmaceutical Benefits Scheme? (e.g. mental health apps vs. antidepressants) - Regulation/guidelines around the prescribing of digital health technologies? | Gauge practitioner perspectives on the need for policies to regulate digital health interventions and the different levels of regulation required. |
|  | **How confident would you be in recommending digital sleep health technologies to patients within the scope of your current clinical practice?** | - Key points to discuss with patients - Key concerns around making recommendations? - Components or functions to expect in sleep DHI? - The importance of f2f care and therapeutic alliance? - Perceived level of upskilling and training required to become confident? - Is the learning curve associated with becoming familiar with a digital platform worth it? | Explore the approach practitioners take when recommending digital health/interventions to patients. |
|  | **What is your perceived level of readiness to roll out digital sleep health technologies within the scope of your current clinical practice?** | **Differences between practices?** (if working >1 practice)  Level of readiness:   - Infrastructural readiness (e.g. IT hardware and connections, and software) - Aptitudinal readiness (e.g. depth of skills and capabilities) - Attitudinal readiness (e.g. willingness to use current and future eHealth solutions).   **Ready:**   - Key enabling factors (reimbursement, costs, human resources, staff training, workflow, time, space) - Any program evaluation? - How do you see digital health interventions positioned in your clinic? E.g., standalone, adjunctive to f2f, provided to those on a clinic waitlist? - Clinical integration of data? Sharing data across other health professionals?   **Not ready:**   - Barriers for uptake within current practice (reimbursement, costs, human resources, staff training workflow, time, space) - The level of (tech) support required to implement digital health technologies into practice? - Is there something similar in your practice already e.g. digital health coordinator? - Would you consider including this role as part of your practice and at what capacity? | Explore whether there exists any infrastructure that would facilitate digital sleep health interventions to be implemented in practice. |
|  | **If digital sleep health was embedded into primary care, what would be the optimal pathway for the patient to access this intervention?** | - Who should initiate the “script” for the digital sleep health intervention? (GP vs. Nurse vs. Pharmacist) - New referral pathways - Digital only vs. f2f and digital - Level of support to upskill the prescribing process. - Reimbursement structures - Potential for collaboration (sharing and accessing patient data)with other health care professionals both in and outside of primary care. | Practitioner perspectives of where digital sleep health technologies can be positioned within current health structures that they operate in, informing a potential conceptual model of care for embedding digital sleep health interventions. |
