# Supplementary material for: Embedding digital sleep health into primary care practice: A triangulation of perspectives from general practitioners, nurses, and pharmacists
Source: Digit Health. 2023 Jun 23;9:20552076231180970. doi: 10.1177/20552076231180970 (PMC10291541; doi:10.1177/20552076231180970)
Supplement: sj-docx-3-dhj-10.1177_20552076231180970 - Supplemental material for Embedding digital sleep health into primary care practice: A triangulation of perspectives from general practitioners, nurses, and pharmacists [file sj-docx-3-dhj-10.1177_20552076231180970.docx]

**Appendix. C Comparison of participant characteristics based on interview status**

| **Covariates** | | | **Interviewed**  **(n = 45)** | **Not Interviewed**  **(n=51)** | **p-value** |
| --- | --- | --- | --- | --- | --- |
|  |  |  | **Mean/Median (SD or Range)** | |  |
| ***^⊥^***Age | | | 43**.**6 (46) | 41**.**0 (43) | 0.28 |
| ***^⊥^*** Average Number of Patients per week | | | 85**.**0 (994) | 60**.**0 (1000) | 0.670 |
| ***^⊥^*** DHI familiarity | | | 2**.**0 (4) | 2**.**0 (4) | 0.464 |
| ***^⊥^*** Sleep DHI familiarity | | | 1**.**0 (3) | 0**.**0 (3) | 0.838 |
| **Attributes when considering implementation of DHIs** | ***^⊥^*** Data Security | | 6**.**0 (5) | 5**.**0 (5) | 0.037 |
|  | ***^⊥^*** Privacy Policy | | 6**.**0 (5) | 6**.**0 (5) | 0.637 |
|  | ***^⊥^*** Legal Compliance | | 6**.**0 (4) | 6**.**0 (5) | 0.389 |
|  | ***^⊥^*** Trustworthy repository | | 6**.**0 (2) | 5**.**0 (4) | 0.039 |
|  | ***^⊥^*** Endorsement | | 5**.**0 (4) | 5**.**0 (4) | 0.482 |
|  | ***^⊥^*** Development by Health care professional or clinical bodies | | 5**.**0 (4) | 5**.**0 (3) | 0.552 |
|  | ***^⊥^*** Demonstrated efficacy | | 5**.**0 (3) | 5**.**00 (4) | 0.768 |
|  | ***^⊥^*** Training | | 5**.**0 (3) | 5**.**00 (4) | 0.312 |
|  | ***^⊥^*** Financial incentives for HCP | | 3**.**0 (6) | 4**.**00 (6) | 0.903 |
|  | ***^⊥^*** Cost to patient | | 5**.**0 (4) | 5**.**00 (4) | 0.377 |
|  | | | ***%(n)*** | ***% (n)*** | **p-value** |
| **Gender (female)** | | | 80**.**0 % (36)_a_ | 60**.**8% (31)_b_ | 0**.**041 |
| **Profession** | | **GP** | 37**.**8% (17) _a_ | 37**.**3% (19) _a_ | 0**.**999 |
|  |  | **Nurse** | 31**.**1% (14) _a_ | 31**.**4% (16) _a_ |  |
|  |  | **Pharmacist** | 31**.**1% (14) _a_ | 31**.**4% (16) _a_ |  |
| **Years of Practice** | **0-5 years** | | 20**.**0% (9) _a_ | 29**.**4% (15) _a_ | 0**.**289 |
|  | **6-10 years** | | 22**.**2% (10) _a_ | 25**.**5% (13) _a_ |  |
|  | **11-20 years** | | 22**.**2% (10) _a_ | 17**.**6% (9) _a_ |  |
|  | **21-30 years** | | 11**.**1% (5) _a_ | 17**.**6% (9) _a_ |  |
|  | **More than 30 years** | | 24**.**4% (11) _a_ | 9**.**8% (5) _a_ |  |
| **State of Practice** | **New South Wales** | | 88**.**9% (40) _a_ | 78**.**4% (40) _a_ | 0**.**342 |
|  | **Victoria** | | 4**.**4% (2) _a_ | 11**.**8% (6) _a_ |  |
|  | **ACT** | | 2**.**2% (1) _a_ | 0**.**0% (0) _a_ |  |
|  | **QLD** | | 2**.**2% (1) _a_ | 2**.**0% (1) _a_ |  |
|  | **SA** | | 0**.**0% (0) _a_ | 5**.**9% (3) _a_ |  |
|  | **TAS** | | 2**.**2% (1) _a_ | 2**.**0% (1) _a_ |  |
|  | **WA** | | N/A | N/A |  |
|  | **NT** | | N/A | N/A |  |
| **Employment Status** | **Full time** | | 42**.**2% (19) _a_ | 39**.**2% (20) _a_ | 0**.**955 |
|  | **Part time** | | 44**.**4% (20) _a_ | 47**.**1% (24) _a_ |  |
|  | **Casual** | | 13**.**3% (6) _a_ | 13**.**7% (7) _a_ |  |
| **Geographical Classification of Practice^a^** | **Metropolitan** | | 73**.**3%% (33) _a_ | 70**.**6% (36) _a_ | 0**.**915 |
|  | **Regional** | | 4**.**4% (2) _a_ | 3**.**9% (2) _a_ |  |
|  | **Rural/Remote** | | 20**.**0% (9) _a_ | 23**.**5% (12) _a_ |  |
| **Sleep-related problems encountered in practice** | **Nil** | | 0**.**0% (0) _a_ | 3**.**9% (2) _a_ | 0**.**166 |
|  | **1-5** | | 46**.**7% (21) _a_ | 43**.**1% (22) _a_ |  |
|  | **6-10** | | 33**.**3% (15) _a_ | 27**.**5% (14) _a_ |  |
|  | **11-15** | | 6**.**7% (3) _a_ | 19**.**6% (10) _a_ |  |
|  | **16+** | | 13**.**3% (6) _a_ | 5**.**9% (3) _a_ |  |
| **Use of DHI in practice (yes)** | | | 62**.**2% (28) _a_ | 45**.**1% (23) _a_ | 0**.**093 |
| **Use of sleep DHI in practice (yes)** | | | 33**.**3% (15) _a_ | 21**.**6% (11) _a_ | 0**.**196 |
| **DHI diagnostic (yes)** | | | 53**.**3% (24) _a_ | 56**.**9% (29) _a_ | 0**.**729 |
| **DHI tracking (yes)** | | | 77**.**8% (35) _a_ | 72**.**5% (37) _a_ | 0**.**555 |
| **DHI educational (yes)** | | | 88**.**9% (40) _a_ | 78**.**4% (40) _a_ | 0**.**170 |
| **DHI behavioural (yes)** | | | 84**.**4% (38) _a_ | 74**.**5% (38) _a_ | 0**.**232 |
| ^a^ Categorization based on the Modified Monash Model^24^ whereby MM1=Metropolitan, MM2= Regional and MM3 to MM7 = Rural and Remote  Test for normality based on the Kolmogorov-Smirnov test as the total sample size is >50  *^⊥^* Mann-Whitney U test, medians and ranges are reported | | | | | |
